# Aspirin Compared With Other Anticoagulants for Use as Venous Thromboembolism Prophylaxis in Elective Orthopaedic Hip and Knee Operations: A Narrative Literature Review

**DOI:** 10.7759/cureus.18249

**Published:** 2021-09-24

**Authors:** Olatomiwa Olukoya, Joshua Fultang

**Affiliations:** 1 Neurocritical Care, National Hospital for Neurology and Neurosurgery, London, GBR; 2 Surgery, University of Glasgow, Glasgow, GBR; 3 General Surgery, University Hospital Ayr/University of West of Scotland, Ayr, GBR

**Keywords:** prophylaxis, dvt, vte, anticoagulation, aspirin

## Abstract

Although total hip and knee arthroplasty are effective methods for treating arthritis, they have an associated risk of venous thromboembolism (VTE). To reduce this risk, prophylactic agents including aspirin, low-molecular-weight Heparin, vitamin K antagonists, and direct oral anticoagulants are employed for up to 35 days after surgery. This narrative literature review utilised a systematic approach to critically assess the current evidence surrounding the use of aspirin for VTE prophylaxis compared to anticoagulants.

An advanced multistage electronic search was performed in May 2021 using the OVID/Medline and Embase online libraries to identify available studies relevant to the subject from 1974. Additional studies identified during the review process were also included. The final studies meeting the inclusion criteria were then assessed using the Critical Appraisal Skills Programme tool.

A total of 12 (60%) studies (two meta-analyses, three randomised trials, seven retrospective studies) favoured aspirin over anticoagulants for VTE prophylaxis. A total of 15 (75%) studies (two meta-analyses, three randomised trials, nine retrospective, one matched cohort) reported that aspirin had better bleeding profiles and complication rates, which was statistically significant in seven (46.7%) studies (one randomised trial, six retrospective studies). A total of eight studies (one randomised trial, six retrospective studies, one matched cohort) reported statistically significant results for aspirin. Five (62.5%) studies reported aspirin to be superior for VTE prophylaxis, while seven (87.5%) reported aspirin to be superior in terms of bleeding complications.

The current evidence indicates that aspirin is superior to anticoagulants, in their various iterations, for VTE prophylaxis in terms of their bleeding profiles.

## Introduction and background

Total hip and knee arthroplasty are well tolerated and effective methods for treating arthritis and result in an improved quality of life for patients [1,2]. Approximately 1.2 million hip replacements and 1.5 million knee replacements were performed between 2003 and 2019 in England, Wales, Northern Island, and the Isle of Man [3].

Despite being common and well-tolerated, these procedures are not completely risk-free. A significant and well documented associated risk is that of venous thromboembolism (VTE) due to the duration of the operation, as well as decreased peri-operative mobility [1]. It is estimated that over 1.8 per 1,000 adults develop acute VTE every year [4]. To reduce the risk of VTE, prophylactic agents are employed for up to 35 days after surgery [1], and it is estimated that the incidence of VTE reduces dramatically to 1.3-10% from 40-60% with prophylaxis [5].

Several anticoagulant agents have been introduced over the years as effective thromboprophylaxis following elective joint arthroplasty procedures, and several studies have examined the efficacy of these agents as well as their side effect profiles, including prolonged wound leakage, bleeding, and infection [6]. Clinicians can choose from any of the following agents: aspirin, low-molecular-weight heparin (LMWH), vitamin K antagonists, and direct oral anticoagulants (DOACs) (including direct thrombin inhibitors [DTIs], for example, dabigatran; and factor Xa inhibitors, for instance, rivaroxaban, edoxaban, apixaban, fondaparinux) [7].

The National Institute for Health and Care Excellence recently changed its recommendation to aspirin as the prophylactic agent of choice following total knee arthroplasty (TKA) or total hip arthroplasty (THA), although an intervening period of 10 days with prophylactic LMWH is required before aspirin in THA [1,7]. The American College of Chest Physician guidelines were initially in opposition to the American Academy of Orthopaedic Surgeons guidelines, recommending against the use of aspirin; however, both guidelines moved toward a consensus in 2012 [8,9].

Aspirin is regarded as an effective form of VTE thromboprophylaxis and is highly valued because it does not require monitoring and has a favourable bleeding profile [1,5]. The only caveat to its use is that it has not been licenced for use as thromboprophylaxis but has been endorsed by BOAST [7]. Although there has been an increased uptake of other newer agents, concerns have been raised with regards to the prolonged bleeding profile of these agents as well as problems with wound complications [1].

Recent trials have shown that aspirin is as effective as other agents for VTE prophylaxis and has better bleeding, wound leakage, and hospital readmission rates [5,10]. Therefore, guidelines now recommend aspirin as an option for VTE prophylaxis, especially in patients at an increased risk of bleeding [11].

This narrative literature review aimed to utilise a systematic approach to offer a critical assessment of current evidence with regards to the use of aspirin for VTE prophylaxis compared to anticoagulants, with a particular emphasis on comparative efficacy and side effect profiles.

## Review

Search strategy and study selection

An advanced multistage electronic search was performed in May 2021 using the OVID/Medline and Embase online libraries to identify available studies relevant to the subject from 1974. Further relevant studies identified during the review process were also added. The final papers for inclusion were then assessed using the Critical Appraisal Skills Programme tool [12].

**Figure 1:**
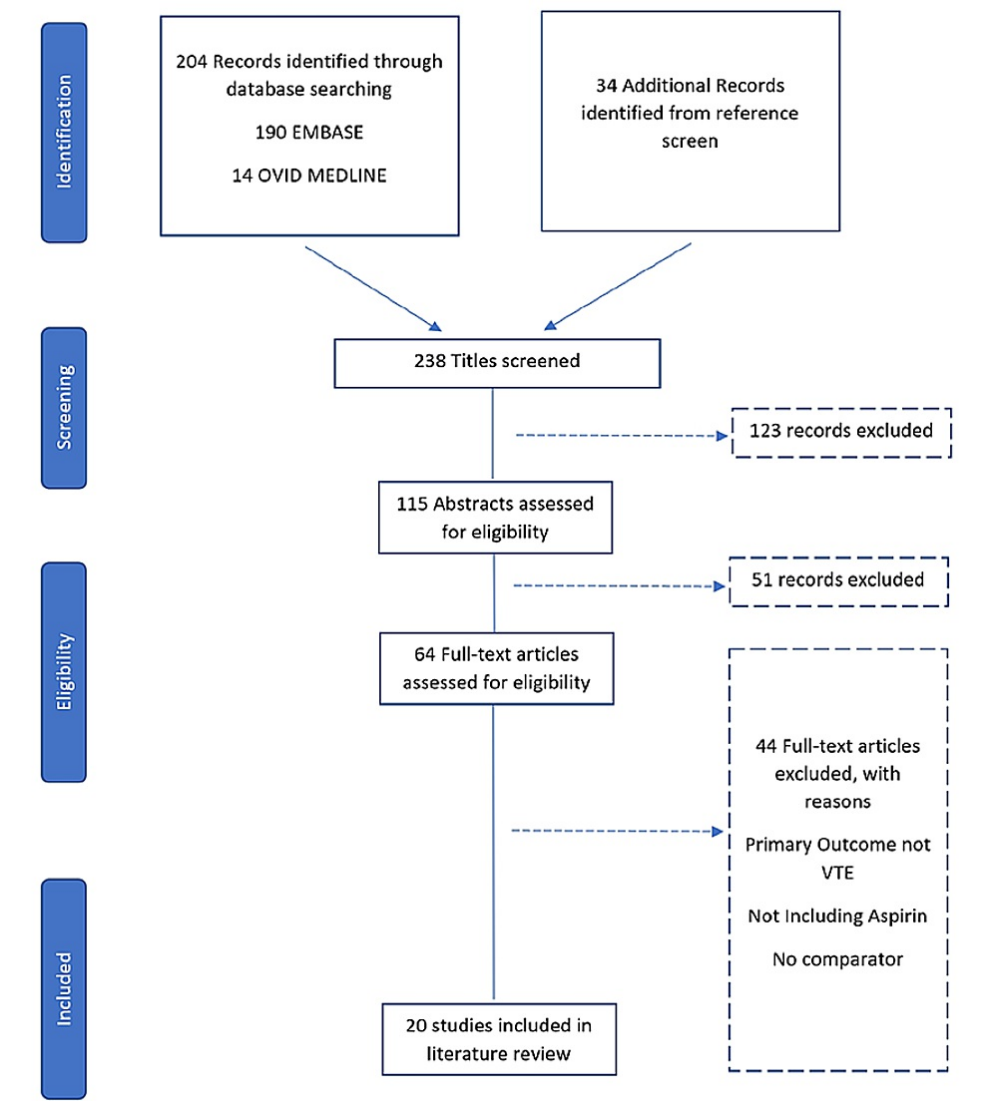
PRISMA diagram. PRISMA flow diagram indicating the number of studies included in this review. The literature search yielded 204 articles, 64 of which were deemed eligible for review and 20 were included in the review. PRISMA: Preferred Reporting Items for Systematic reviews and Meta-Analyses; VTE: venous thromboembolism

Exclusion criteria included all publications with abstracts only, animal studies, case reports, studies not comparing aspirin to other anticoagulants, non-English language, non-orthopaedic procedures, studies examining non-elective procedures, studies looking only at cost-effectiveness, studies not reporting on VTE as an outcome, studies primarily involving non-pharmacological prophylaxis comparators, studies involving dextrans and dihydroergotamine (DHE)-heparin, studies where agents were administered based on risk stratification, studies where cohorts were given a combination of one or more of the comparative drugs sequentially without a clear delineation of the effect between the drugs, and duplicate studies. The main inclusion criteria were studies comparing the efficacy of aspirin for anticoagulation in elective orthopaedic hip and knee operations to other commonly used anticoagulants. Figure 1 shows a detailed Preferred Reporting Items for Systematic reviews and Meta-Analyses (PRISMA) [13] flow diagram.

Several agents have been described in the literature as being effective for VTE prophylaxis following elective hip and knee arthroplasty. These include aspirin, vitamin K antagonists (warfarin), direct thrombin inhibitors (DTIs) (dabigatran), LMWH (enoxaparin), and factor Xa inhibitors (rivaroxaban, apixaban, edoxaban, fondaparinux) [7]. Table 1 outlines a summary of the comparative efficacy of these agents as described in the literature.

**Table 1:** Comparative efficacy of the various prophylactic agents The most effective VTE agent has been assigned a numerical value of 1, with an increasing number denoting a comparatively lower efficacy. The most effective agents in terms of bleeding profile and/or complications have been assigned an alphabetic character “A,” with subsequent letters denoting a comparatively lower efficacy when multiple agents are compared. A star (*) has been used to denote if the result is statistically significant. Duplication of an alphanumeric character indicates comparable efficacy or that the study did not make any distinction between the agents. THA = total hip arthroplasty; TKA = total knee arthroplasty; TJA = total joint arthroplasty; RCT = randomised controlled trial

Author	Year		Study	Operation	No.	Aspirin	Warfarin	Enoxaparin/LMWH	DOACs (Factor Xa inhibitors and DTIs)						
									Dabigatran (DTI)	Rivaroxaban	Apixaban	Factor Xa	Edoxaban	Pentasaccharides (fondaparinux/idraparinux)	Not specified
Matharu et al. [1]		2020	Meta-analysis	THA, TKA	6,060	1-	2-	2-		2-					
Hasan et al. [5]		2021	Retrospective	TKA	420	2A					1B				
Drescher et al. [10]		2014	Meta-analysis	THA, TKA	1,408	2A	1B	1B							1B
Chu et al. [11]		2017	Retrospective	THA, TKA	342, 401	(1A)*	(2B)*	(2B)*	(2B)*	(2B)*	(2B)*	(2B)*		(2B)*	
Anderson et al. [14]		2018	RCT	THA, TKA	3,424	1B				2A					
Colleoni et al. [15]		2018	RCT	TKA	32	1A				2B					
Matharu et al. [16]		2020	Matched cohort	THA, TKA	218, 650	3*A			2*A			1*A			
Brown et al. [17]		2009	Meta-analysis	THA, TKA	34,847	1A	4C	2D						3B	
Bala et al. [18]		2017	Retrospective	THA	18, 288	(1A)*	(4C)*	(3D)*		(2B)*	(2B)*	(2B)*		(2B)*	
Bala et al. [19]		2020	Retrospective	THA	8,829	(1A)*	(4C)*	(3D)*		(2B)*	(2B)*	(2B)*		(2B)*	
Hood et al. [20]		2019	Retrospective	TKA	41,537	(1A)*	(2B)*	(2B)*				(2B)*			
Bawa et al. [21]		2018	Retrospective	THA, TKA	239, 949	2-	6-	5-	4-	1-				3-	
Raphael et al. [22]		2014	Retrospective	TJA	28, 923	1A*	2B								
Huang et al. [23]		2016	Retrospective	TJA	30,270	(1A)*	(2B)*								
Lotke et al. [24]		1996	RCT	THA, TKA	312	2-	1-								
Jameson et al. [25]		2011	Retrospective	THA	108, 584	2B		1A							
Jameson et al. [26]		2012	Retrospective	TKA	156, 798	2A		1B							
Anderson et al. [27]		2013	RCT	THA	778	1A		2B							
Zou et al. [28]		2014	RCT	TKA	324	(3A)*		(2B)*		(1C)*					
Nielen et al. [29]		2016	Retrospective	TKA, THA	7,101	1A		3C							2B

Aspirin versus direct oral anticoagulants

DOACs encompass a broad range of medications across two major drug classes: DTIs and factor Xa inhibitors. The literature search identified four studies comparing aspirin to DOACs in their various iterations.

A large double-blind randomised control trial (n = 3,424) by Anderson et al. (2018) [14], with a follow-up of 90 days, found no difference in the prophylactic efficacy of aspirin when compared to rivaroxaban. They noted an incidence of VTE of 0.64% in the aspirin group compared to 0.70% in the rivaroxaban group (95% confidence interval [CI] = 0.55-0.66). However, they found the incidence of major and non-major bleeding to be higher in the aspirin group compared to the rivaroxaban group (1.29% vs. 0.99%; p-value = 0.43). This was, however, confounded by the fact that some trial participants were permitted to continue their usual dose of aspirin in addition to the randomised prophylaxis protocol.

A small single-centre randomised study (n = 32) by Colleoni et al. (2018) [15] with a follow-up period of four weeks, although reporting no statistically significant results, demonstrated a slightly higher incidence of deep vein thrombosis (DVT) in those given a DOAC (Rivaroxaban 10 mg for 14 days) compared with aspirin (300 mg in two divided doses) (11.1% vs. 7.1%; p-value = 1). Additionally, the researchers found that wound dehiscence rates were higher in the DOAC group compared to the aspirin group (16.7% vs. 7.1%). Similarly, the reoperation rates and incidence of death were higher in the DOAC group (reoperation: 11.1% vs. 7.1%; p-value = 1; death: 5.6% vs. 0%; p-value = 1). Interestingly, however, there was a slightly higher rate of hospital readmission in the aspirin group compared with the DOAC group (14.3% vs. 11.1%; p-value = 1).

A large retrospective matched cohort study (n = 218,650) by Matharu et al. (2020) [16] which looked at patients over a 15-year period found that after elective total hip and knee arthroplasty, DTIs, and factor Xa Inhibitors had a significantly lower risk of VTE compared with aspirin (THA: DTI vs. aspirin: 0.44% vs. 0.63%; odds ratio [OR] = 0.69; 95% CI = 0.55-0.87; p-value = 0.002; THA: factor Xa vs. aspirin: 0.37% vs. 0.59%; OR = 0.63; 95% CI, 0.47-0.85; p-value = 0.003; TKA: DTI vs. aspirin: 0.60% vs. 0.73%; OR = 0.82; 95% CI = 0.68-0.98; p-value = 0.032; TKA: factor Xa vs. aspirin: 0.49% vs. 0.68%; OR = 0.73; 95% CI = 0.58-0.91; p-value = 0.006). They also established that DTIs and factor Xa inhibitors were superior to aspirin in terms of patient length of stay in hospital and readmission rates. However, there was no difference between aspirin and DOACs in the main adverse events of wound complication and revision surgery [14].

In contrast, a single-centre retrospective study by Hasan et al. (2021) [5] suggested that aspirin was marginally better than apixaban with regards to wound leakage, although the result was not statistically significant (6.0% wound leakage rate with apixaban vs. 5.3% with aspirin; p-value = 0.325). However, they reported that apixaban resulted in a lower incidence of VTE compared to aspirin, although this was not statistically significant (0.7% vs. 2.4%; p-value = 0.152). The 30-day readmission rate was higher in those given aspirin than those given apixaban. Both aspirin and apixaban were equally safe with regard to major bleeding risk.

The above evidence shows that aspirin has a comparable VTE prophylactic efficacy and bleeding profile compared to DOACs. Of note, studies with higher levels of evidence suggested that aspirin had a slight advantage.

Aspirin versus warfarin, enoxaparin, and direct oral anticoagulants

Several studies, cognizant of the variety of thromboprophylaxis agents available, sought to compare the efficacy of aspirin to warfarin, enoxaparin, and factor Xa inhibitors. The literature search identified eight studies comparing aspirin to this broader range of anticoagulants.

A recent meta-analysis of 13 randomised control trials comparing aspirin to anticoagulants, such as LMWH, rivaroxaban, warfarin, dextrans and DHE-heparin, by Matharu et al. (2020), although reporting no statistically significant results, found that most studies were in favour of aspirin for VTE prophylaxis [1]. However, some studies [1] in the meta-analysis included non-pharmacological prophylactic measures as comparators and did not meet all the inclusion criteria for a separate review in this article. The bleeding risk between the agents was not statistically significant; however, the analysis made no mention of whether most studies favour one agent over the other in terms of clinical significance [1].

Another meta-analysis by Drescher et al. (2014) [10] examining eight randomised control trials, comparing aspirin to warfarin, LMWH, heparin and danaparoid, showed that the incidence of DVTs and pulmonary embolisms (PEs) was lower in the anticoagulation group compared to the aspirin group, although this finding was not statistically significant. However, they reported a reduced risk of bleeding in those given aspirin as opposed to anticoagulation. This finding was statistically significant in two studies looking at non-elective operations, but not statistically significant in the arthroplasty groups.

A slightly older meta-analysis by Brown et al. (2009) [17] pooled data from 14 randomised controlled trials which assessed the risk of VTE and bleeding risk with aspirin, LMWH, fondaparinux, warfarin, and placebo. They showed that aspirin had a lower average incidence of VTE, followed by LMWH, fondaparinux, and warfarin. However, warfarin was found to have the lowest incidence of fatal PE. Aspirin had the lowest incidence of operative site bleeding, followed by fondaparinux, with LMWH being the worst. The study, however, did not compare aspirin to other agents as a whole category, rather opting to compare them separately: aspirin versus fondaparinux; aspirin versus LMWH; and aspirin versus warfarin. They ultimately concluded that there was no statistically significant difference in clinically relevant VTE outcomes, while the anticoagulants increased the risk of operative site bleeding.

Another large retrospective study [18], with a 90-day follow-up period, compared the efficacy of these drugs after TKA and found that aspirin and factor Xa inhibitors had the lowest incidence of DVT and PE from two weeks to 90 days, with enoxaparin being slightly worse and warfarin being the worst (p-value < 0.01). In terms of major bleeding risk requiring transfusion, aspirin had the lowest incidence closely followed by factor Xa inhibitors, with enoxaparin performing worse than warfarin (p-value < 0.01). Bala et al. published an updated study in 2019 [19] and examined the efficacy of the same medications following THA with results comparable to the findings presented in their earlier study [18].

A recent large retrospective cohort study (n = 41,537) by Hood et al. (2019) [20] found Aspirin to have a lower incidence of VTE compared to anticoagulation (warfarin, LMWH, and factor Xa inhibitors) (3.1% vs. 6.8%; p < 0.001). Moreover, multivariable analyses showed aspirin to be comparable to anticoagulants. The authors demonstrated similar findings with regards to the risk of bleeding, with aspirin having a slightly lower risk of bleeding compared to anticoagulants (0.90% vs. 1.14%; p-value < 0.01).

A recent retrospective review of a claims database over 10 years, comprising 20% of the insurance market in the United States, for aspirin, warfarin, enoxaparin, fondaparinux, rivaroxaban, and dabigatran by Bawa et al. (2018) [21] found that the incidence of DVT was the lowest in the rivaroxaban group, followed by the aspirin and fondaparinux groups and the highest in the warfarin, enoxaparin, and dabigatran groups (1.86% for rivaroxaban, 2.20% for aspirin, and 2.69% for fondaparinux versus 4.74%, 3.83%, and 3.73%, respectively) [21]. This finding was upheld by a multivariate regression analysis adjusting for confounders, confirming that patients prescribed aspirin, fondaparinux, and rivaroxaban were less likely to have a DVT (OR = 0.69, 95% CI = 0.49-0.96 for aspirin; OR = 0.85, 95% CI = 0.76-0.95 for fondaparinux; and OR = 0.57, 95% CI = 0.51-0.63 for rivaroxaban) compared to those given warfarin, enoxaparin, or dabigatran (OR = 3.60, 95% CI = 3.38-3.84 for warfarin; odds ratio = 1.14, 95% CI = 1.09-1.20 for enoxaparin; and OR = 1.09, 95% CI = 0.80-1.47 for dabigatran). However, this study did not compare these medications based on adverse events, namely, major bleeding or wound complications.

A large retrospective cohort study in the United States (n = 342,401), based on a database of over 700 small-to-moderately sized hospitals by Chu et al. (2017) [11] demonstrated that the rates of VTE and haemorrhagic complications were the lowest in the aspirin-only cohort compared to the anticoagulant-only or a combination regime, although the finding was only statistically significant in those who underwent TKA.

Most of the studies concur that aspirin is superior to this broader category of anticoagulants in terms of prophylaxis and bleeding profile, with two out of the three meta-analyses declaring aspirin the clear favourite.

Aspirin versus warfarin

Warfarin was the preferred anticoagulation of choice before the advent of DOACs. It was, however, associated with an increased risk of operative site bleeding as well as requiring therapeutic monitoring. Several studies have sought to establish if aspirin is an efficacious substitute with fewer drawbacks.

A large retrospective cohort study by Raphael et al. [22] found aspirin to be superior to warfarin in the prevention of symptomatic DVT and PE. This finding was statistically significant and persisted even after propensity matching: 0.11% symptomatic PE incidence in the aspirin group compared with 0.67% in the warfarin group (OR = 6.36, 95% CI = 1.64-54.50; p-value = 0.002); 0.11% symptomatic DVT rate in the aspirin group compared with 0.91% in the warfarin group (OR = 8.57, 95% CI = 2.25-72.58; p-value <0.001). Similar to other published literature, Raphael et al. [22] also demonstrated that aspirin was superior to warfarin in terms of wound-related complications (bleeding and wound drainage); however, only the wound drainage finding was statistically significant after propensity score matching (p-value = 0.016).

A large retrospective study by Huang et al. [23] (n = 30,270) adopted a slightly different approach to most other studies, opting to compare the efficacy of these two medications in patients at a higher risk of VTE. This study met the inclusion criteria as the prophylactic agents were administered prospectively, irrespective of the risk status; the researchers classified subjects retrospectively as either high or low risk. They found that the incidence of VTE was statistically significantly lower in the aspirin group compared with Warfarin, irrespective of the risk status (p < 0.001). Similarly, they found aspirin to be superior in terms of would complications and bleeding risk.

In contrast, a randomised control trial by Lotke et al. (1996) [24], paying careful attention to investigating the size, incidence, and location of VTE via venograms and ventilation-perfusion scans, found the incidence of VTE to be higher in the aspirin group compared to the Warfarin group, although this was not a statistically significant difference. The researchers did not, however, compare the two agents based on their haemorrhagic profile.

The consensus of the literature is that aspirin is superior to warfarin with regards to its thromboprophylaxis profile as well as bleeding profile, with the two large retrospective studies reporting statistically significant results despite not being randomised.

Aspirin versus low-molecular-weight heparin

LMWH is now routinely used in most hospitals as an effective thromboprophylaxis agent in both medical and surgical patients. The concern with this agent is regarding complications with bleeding risk. The literature search identified three studies comparing aspirin with LMWH.

The first of these studies, a large retrospective cohort study (n = 108,584) by Jameson et al. (2011) [25] examining the use of these medications following THA, although reporting no statistically significant differences, found the incidence of VTE to be higher in the aspirin group compared to the LMWH group following propensity score matching. However, in contrast to other studies [26-29], they found the rate of haemorrhage and mortality to be higher in the aspirin group compared to the LMWH group; however, the rate of reoperation was the highest in the LMWH group.

The same group of researchers performed a similar study in 2012 [26] in which they evaluated the comparative efficacy of these medications following TKA. They again demonstrated that the rate of VTE was higher in the aspirin cohort than the warfarin cohort. However, in this study, they noticed a lower incidence of haemorrhage and mortality in the aspirin cohort. These findings, were, however, not statistically significant. The rate of reoperation was greater in the aspirin cohort, and this finding was statistically significant (p-value = 0.01).

A randomised control trial by Anderson et al. [27] investigated the same drugs. Their study had to be terminated before reaching a sufficient number to potentially demonstrate the superiority of one over the other due to a change in policy resulting in a switch to rivaroxaban. However, the interim analysis showed that they had sufficient power to demonstrate the non-inferiority of one drug over the other. Their study went on to show a lower rate of VTE in the aspirin cohort compared with LMWH. Moreover, the results showed that aspirin was non-inferior, but not superior, to LMWH (p-value < 0.001 for non-inferiority; p-value = 0.22 for superiority). When comparing these medications in terms of bleeding complications, they found a lower incidence with aspirin compared with LMWH; however, this did not reach statistical significance.

The consensus of the literature appears to favour LMWH as being the better thromboprophylaxis agent; however, the consensus is that aspirin has a better bleeding profile. However, it is important to bear in mind that none of the studies reported statistically significant results.

Aspirin versus low-molecular-weight heparin and direct oral anticoagulants

As noted in the randomised study by Anderson et al. [27], rivaroxaban was introduced as an agent for VTE prophylaxis instead of LMWH leading to early termination of their study. Several studies, aware of this new medication, sought to compare the efficacy of both rivaroxaban and LMWH and aspirin due to issues surrounding haemorrhagic complications with anticoagulant agents. The literature search identified three studies dealing primarily with these three drugs.

Zou et al. (2014) [28] conducted a randomised control trial in China examining the comparative efficacy of the aforementioned three medications. They found that the incidence of VTE was the lowest in the rivaroxaban group, followed by the LMWH cohort and the highest in the aspirin cohort. Rivaroxaban was statistically significantly better at reducing VTE compared to LMWH and aspirin (p-value = 0.029 and p-value = 0.017, respectively); however, there was no statistically significant difference between aspirin and LMWH. As predicted by the investigators, haemorrhagic complications (hidden blood loss) were the highest in the rivaroxaban cohort and the lowest in the LMWH cohort. The incidence of subcutaneous ecchymoses, however, was the lowest in the aspirin cohort. This was statistically significant only when comparing rivaroxaban to the other two agents, but not when comparing aspirin to LMWH.

In contrast, a large retrospective cohort study by Nielen et al. [29] (n = 7,101) found Aspirin to have the lowest incidence of VTE prophylaxis, averaging over THA and TKA, with LMWH having the highest incidence. The average incidence of haemorrhagic complications was similar. However, their study did not report statistically significant results.

The literature, although not reporting any statistically significant results, is split on the comparative efficacy of aspirin to the anticoagulants, LMWH and DOACs; however, there is a strong consensus regarding the superiority of aspirin in terms of its bleeding and complications profile.

## Conclusions

The current evidence indicates that aspirin is superior to anticoagulants, in their various iterations, for VTE prophylaxis and in terms of their bleeding profiles. Out of the 20 studies, 12 (60%) included favoured aspirin over anticoagulants for VTE prophylaxis, although only in five (41.7%) of those studies was the finding statistically significant. Similarly, out of the 20 studies, 15 (75%) demonstrated the superiority of aspirin to anticoagulants in terms of their bleeding profiles and complication rates, although the findings were only statistically significant in seven (46.7%) of the studies. When examining the statistically significant results for aspirin, eight studies were identified. One study reported only statistical significance for VTE prophylaxis and another reported significance only for bleeding complications. Of these studies, five (62.5%) reported the superiority of aspirin to anticoagulants in terms of their efficacy for VTE prophylaxis, while seven (87.5%) declared aspirin superior in terms of bleeding complications. Therefore, it is evident that the superiority of aspirin to anticoagulants is both clinically and statistically significant.

## References

[1] Matharu GS, Kunutsor SK, Judge A, Blom AW, Whitehouse MR (2020). Clinical effectiveness and safety of aspirin for venous thromboembolism prophylaxis after total hip and knee replacement: a systematic review and meta-analysis of randomized clinical trials. JAMA Intern Med.

[2] Learmonth ID, Young C, Rorabeck C (2007). The operation of the century: total hip replacement. Lancet.

[3] (2021). National Joint Registry Report. Joint replacement statistics. https://reports.njrcentre.org.uk/Portals/0/PDFdownloads/NJR%2017th%20Annual%20Report%202020.pdf.

[4] Sobieraj DM, Coleman CI, Pasupuleti V, Deshpande A, Kaw R, Hernandez AV (2015). Comparative efficacy and safety of anticoagulants and aspirin for extended treatment of venous thromboembolism: a network meta-analysis. Thromb Res.

[5] Hasan SS, Sunter W, Ahmed N, Dawoud D, Zaidi ST (2021). Venous thromboembolism prophylaxis in patients undergoing knee replacements: comparison of real-world outcomes. Int J Clin Pharm.

[6] Garfinkel JH, Gladnick BP, Roland N, Romness DW (2018). Increased incidence of bleeding and wound complications with factor-Xa inhibitors after total joint arthroplasty. J Arthroplasty.

[7] National Institute for Health and Care Excellence (NICE). (2021). National Institute for Health and Care Excellence (NICE). Venous thromboembolism in over 16s: reducing the risk of hospital-acquired deep vein thrombosis or pulmonary embolism. NICE Guideline.

[8] An VV, Phan K, Levy YD, Bruce WJ (2016). Aspirin as thromboprophylaxis in hip and knee arthroplasty: a systematic review and meta-analysis. J Arthroplasty.

[9] Intermountain Joint Replacement Centre Writing Committee (2012). A prospective comparison of warfarin to aspirin for thromboprophylaxis in total hip and total knee arthroplasty. J Arthroplasty.

[10] Drescher FS, Sirovich BE, Lee A, Morrison DH, Chiang WH, Larson RJ (2014). Aspirin versus anticoagulation for prevention of venous thromboembolism major lower extremity orthopedic surgery: a systematic review and meta-analysis. J Hosp Med.

[11] Chu JN, Maselli J, Auerbach AD, Fang MC (2017). The risk of venous thromboembolism with aspirin compared to anticoagulants after hip and knee arthroplasty. Thromb Res.

[12] Brice Brice, R. R. (2021). CASP Checklists. https://casp-uk.net/casp-tools-checklists.

[13] Page MJ, McKenzie JE, Bossuyt PM (2021). The PRISMA 2020 statement: an updated guideline for reporting systematic reviews. BMJ.

[14] Anderson DR, Dunbar M, Murnaghan J, Kahn SR, Gross P, Forsythe M (2018). Aspirin or rivaroxaban for VTE prophylaxis after hip or knee arthroplasty. J Vasc Surg.

[15] Colleoni JL, Ribeiro FN, Mos PA, Reis JP, Oliveira HR, Miura BK (2018). Venous thromboembolism prophylaxis after total knee arthroplasty (TKA): aspirin vs. rivaroxaban. Rev Bras Ortop.

[16] Matharu GS, Garriga C, Whitehouse MR, Rangan A, Judge A (2020). Is aspirin as effective as the newer direct oral anticoagulants for venous thromboembolism prophylaxis after total hip and knee arthroplasty? An analysis from the National Joint Registry for England, Wales, Northern Ireland, and the Isle of Man. J Arthroplasty.

[17] Brown GA (2009). Venous thromboembolism prophylaxis after major orthopaedic surgery: a pooled analysis of randomized controlled trials. J Arthroplasty.

[18] Bala A, Huddleston JI 3rd, Goodman SB, Maloney WJ, Amanatullah DF (2017). Venous thromboembolism prophylaxis after TKA: aspirin, warfarin, enoxaparin, or factor Xa inhibitors?. Clin Orthop Relat Res.

[19] Bala A, Murasko MJ, Burk DR, Huddleston JI 3rd, Goodman SB, Maloney WJ, Amanatullah DF (2020). Venous thromboprophylaxis after total hip arthroplasty: aspirin, warfarin, enoxaparin, or factor Xa inhibitors?. Hip Int.

[20] Hood BR, Cowen ME, Zheng HT, Hughes RE, Singal B, Hallstrom BR (2019). Association of aspirin with prevention of venous thromboembolism in patients after total knee arthroplasty compared with other anticoagulants: a noninferiority analysis. JAMA Surg.

[21] Bawa H, Weick JW, Dirschl DR, Luu HH (2018). Trends in deep vein thrombosis prophylaxis and deep vein thrombosis rates after total hip and knee arthroplasty. J Am Acad Orthop Surg.

[22] Raphael IJ, Tischler EH, Huang R, Rothman RH, Hozack WJ, Parvizi J (2014). Aspirin: an alternative for pulmonary embolism prophylaxis after arthroplasty?. Clin Orthop Relat Res.

[23] Huang RC, Parvizi J, Hozack WJ, Chen AF, Austin MS (2016). Aspirin is as effective as and safer than warfarin for patients at higher risk of venous thromboembolism undergoing total joint arthroplasty. J Arthroplasty.

[24] Lotke PA, Palevsky H, Keenan AM, Meranze S, Steinberg ME, Ecker ML, Kelley MA (1996). Aspirin and warfarin for thromboembolic disease after total joint arthroplasty. Clin Orthop Relat Res.

[25] Jameson SS, Charman SC, Gregg PJ, Reed MR, van der Meulen JH (2011). The effect of aspirin and low-molecular-weight heparin on venous thromboembolism after hip replacement: a non-randomised comparison from information in the National Joint Registry. J Bone Joint Surg Br.

[26] Jameson SS, Baker PN, Charman SC, Deehan DJ, Reed MR, Gregg PJ, Van der Meulen JH (2012). The effect of aspirin and low-molecular-weight heparin on venous thromboembolism after knee replacement: a non-randomised comparison using National Joint Registry Data. J Bone Joint Surg Br.

[27] Anderson DR, Dunbar MJ, Bohm ER (2013). Aspirin versus low-molecular-weight heparin for extended venous thromboembolism prophylaxis after total hip arthroplasty: a randomized trial. Ann Intern Med.

[28] Zou Y, Tian S, Wang Y, Sun K (2014). Administering aspirin, rivaroxaban and low-molecular-weight heparin to prevent deep venous thrombosis after total knee arthroplasty. Blood Coagul Fibrinolysis.

[29] Nielen JT, Dagnelie PC, Emans PJ (2016). Safety and efficacy of new oral anticoagulants and low-molecular-weight heparins compared with aspirin in patients undergoing total knee and hip replacements. Pharmacoepidemiol Drug Saf.

